# Generation of potent cytotoxic T lymphocytes against in male patients with non-muscle invasive bladder cancer by dendritic cells loaded with dying T24 bladder cancer cells

**DOI:** 10.1590/S1677-5538.IBJU.2016.0274

**Published:** 2017

**Authors:** Eu Chang Hwang, Seung Il Jung, Hyun-Ju Lee, Je-Jung Lee, Dong Deuk Kwon

**Affiliations:** 1 Research Center for Cancer Immunotherapy, Chonnam National University Hwasun Hospital, Hwasun, Jeollanamdo, Republic of Korea;; 2 Department of Urology, Chonnam National University Hwasun Hospital, Hwasun, Jeollanamdo, Republic of Korea; 3 Department of Hematology-Oncology, Chonnam National University Hwasun Hospital, Hwasun, Jeollanamdo, Republic of Korea;; 4 Vaxcell-Bio Therapeutics, Hwasun, Jeollanamdo, Republic of Korea;; 5 The Brain Korea 21 Project, Center for Biomedical Human Resources at Chonnam National University, Gwangju, Republic of Korea

**Keywords:** Dendritic Cells, Urinary Bladder Neoplasms, T-Lymphocytes, Cytotoxic

## Abstract

**Background:**

In order to induce a potent cytotoxic T lymphocyte (CTL) response in dendritic cell (DC)-based immunotherapy for bladder cancer, various tumor antigens can be loaded onto DCs.

**Objective:**

The aim of this study was to establish a method of immunotherapy for male patients with non-muscle invasive bladder cancer (NMIBC), using bladder cancer-specific CTLs generated in vitro by DCs.

**Materials and Methods:**

Monocyte-derived DCs from bladder cancer patients were induced to mature in a standard cytokine cocktail (IL-1β, TNF-α, IL-6, and PGE_2_: standard DCs, sDCs) or anα-type 1-polarized DC (αDC1) cocktail (IL-1β, TNF-α, IFN-α, IFN-γ, and polyinosinic:polycytidylic acid) and loaded with the UVB-irradiated bladder cancer cell line, T24. Antigen-loaded αDC1s were evaluated by morphological and functional assays, and the bladder cancer-specific CTL response was analyzed by cytotoxic assay.

**Results:**

The αDC1s significantly increased the expression of several molecules pertaining to DC maturation, regardless of whether or not the αDC1s were loaded with tumor antigens, relative to sDCs. The αDC1s demonstrated increased production of interleukin-12 both during maturation and after subsequent stimulation with CD40L that was not significantly affected by loading with tumor antigens as compared to that of sDCs. Bladder cancer-specific CTLs targeting autologous bladder cancer cells were successfully induced by αDC1s loaded with dying T24 cells.

**Conclusion:**

Autologous αDC1s loaded with an allogeneic bladder cancer cell line resulted in increased bladder cancer-specific CTL responses as compared to that with sDCs, and therefore, may provide a novel source of DC-based vaccines that canbe used in immunotherapy for male patients with NMIBC.

## INTRODUCTION

Urothelial carcinoma (UC) can be defined as neoplasms that arise from the epithelial lining of the urinary tract, from the minor calyces to the urinary bladder and even to the prostatic urethra. Based on histological evidence, majority of UC is bladder cancer (1). UC accounts for 90% of bladder tumors, of which approximately 70% are confined to layers above the muscularis propria; these comprise the so-called non-muscle invasive bladder cancer (NMIBC). These tumors (previously termed “superficial bladder tumors”) include stages Ta, T1, and Tis, which occur in 70, 20, and 10% of NMIBC cases, respectively (2). Standard primary treatment for NMIBC is transurethral resection; however, a problem in the management of NMIBC is its high intravesical recurrence rate, which ranges from 30 to nearly 80%, depending on the risk profile. Several mechanisms for intravesical recurrence have been proposed, including microscopic persistence of tumor, cancer cell implantation, and new tumor formation (1). More importantly, NMIBC may progress to muscle-invasive cancer during repeated episodes of intravesical recurrence. High rates of recurrence and progression of NMIBC have prompted investigation into a myriad of treatments attempting to decrease the burden of this tumor. Typically, treatment for high-risk NMIBC involves transurethral resection of the bladder tumor, and subsequent adjuvant therapy with Bacillus Calmette-Guerin (BCG) as one of feasible choices. The precise immunological mechanism of BCG has not been determined, but it is assumed that BCG is dependent on T cells. The role of Th1-mediated immunity, including CD4+ T cells and CD8+ cytotoxic T lymphocytes (CTLs), is well known. Ratliff et al.(3) showed that athymic nude mice did not undergo BGC-mediated antitumor activity. BCG treatment can reduce the risk of recurrence and progression of NMIBC, and is regarded as the most successful immunotherapy to date (4). However, 30-45% of patients are BCG failures, and its use is limited by its adverse effect profile and an intolerance that occurs in 20% of patients (5). Thus, new effective bladder-sparing treatments are needed in patients with NMIBC following BCG failure.

Dendritic cells (DCs) have the unique capacity to establish a primary immune response against tumor-associated antigens (TAA). This essential role of DCs in cellular immunity has led to the development of feasible and effective DC-based vaccines against tumor antigens to eliminate tumor cells (6). As a result, clinical trials using DC-based immunotherapeutic targeting of tumors are now underway (7).

Previously, Jonuleit et al.(8) introduced sDCs induced by a cytokine cocktail containing tumor necrosis factor (TNF), α/interleukin (IL)-1β/IL-6, and prostaglandin E_2_ (PGE_2_),which have been used in several clinical studies (9). However, the main disadvantage of sDCs is the absence of IL-12p70 secretion(10).This is important for the induction of effective Th1 and cytotoxic T lymphocyte (CTL) responses, which are assumed to be essential to cancer vaccination therapy. In an attempt to increase the potency of DCs, αDC1s were developed through the use of cytokine combinations. The αDC1s are induced to mature by the addition of an αDC1-polarizing cytokine cocktail containing IL-1β, TNF-α, IFN-α, IFN-γ, and polyinosinic:polycytidylic acid [poly(I:C)]. Compared to sDCs, αDC1s generate stronger and more functional CTLs for several diseases (11).

Effective tumor antigens are another important consideration of DC-based immunotherapy. Several studies have demonstrated that DCs pulsed with peptide and genescan generate potent bladder cancer-specific CTLs (12,13). The use of whole tumor cells rather than single antigens has the advantage of targeting multiple tumor antigens at once, which should avoid antigen escape mechanisms and allow for effective targeting of the majority of tumor cells within a growing tumor. However, this approach is technically limited, because harvesting tumor cells and generating a vaccine line that expresses a standardized amount of cytokine is not always feasible. It can be expensive, time-consuming, and unsuitable for those with a lower tumor burden status (e.g., carcinoma in situ).

To overcome this limitation, allogeneic tumor cells or established cancer cell lines from various tumors have been used as an alternative source of tumor-relevant antigens (14). Vaccines made from allogeneic cells circumvent the issue of individualizing each patient’s therapy, and by using several cell lines derived from different tumors in the vaccine, there is an increased likelihood that the patient’s tumor will share antigens expressed by the vaccine cells, including important tumor antigens that are frequently overexpressed or mutated in that particular cancer (15).

In the present study, we investigated the feasibility of DC-based immunotherapy for male patients with NMIBC. To achieve this purpose, αDC1s were loaded with UVB-irradiated allogeneic bladder cancer cells as tumor antigens. Their ability to elicit specific immune responses mediated by CTLs in vitro relative to sDCs loaded with tumor antigens was then assessed by IFN-γ enzyme-linked immunospot (ELISPOT) assay.

## MATERIALS AND METHODS

### Patient characteristics

A total of five patients with initial NMIBC treated by transurethral resection were enrolled for blood and tumor cell sampling. Patient demographics are shown in Table-1.

**Table 1:** Patient demographics.

	Sex	Age	Tumor size	Histologic examination
Patient 1	Male	72	1.5cm	T1 high grade, CIS
Patient 2	Male	74	2cm	T1 low grade
Patient 3	Male	77	2.5cm	T1 high grade
Patient 4	Male	74	1.5cm	Ta high grade
Patient 5	Male	55	1.5cm	T1 low grade

The study protocol was approved by the institutional review board at the Chonnam National University Hwasun Hospital (IRB registration number-2010-88; Hwasun, Korea). Informed consent was obtained from each patient.

### Generation of αDC1s from patients with bladder cancer

Peripheral blood mononuclear cells were collected from bladder cancer patients. CD14+monocytes were isolated by positive selection using the magnetic-activated cell sorting system (MACS; Miltenyi Biotec, Auburn, CA, USA). The purity of the CD14+ cells was >90%. To generate immature DCs (iDCs), CD14+monocytes were cultured in IMDM (Gibco-BRL, Seoul, Korea) with 10% heat-inactivated FBS (Hyclone) and 1% penicillin/streptomycin (Gibco-BRL) for 6 d in 24-well plates with 5×10^5^ cells per well in the presence of 50ng/mL granulocyte-macrophage colony-stimulating factor (GM-CSF) (PEPROTECH, Rocky Hill, NJ) and 20ng/mL IL-4 (PEPROTECH). On day 6, the iDCs were matured with either conventional cytokine cocktail composed of IL-1ß (25ng/mL, PEPROTECH), TNF-α (50ng/mL, PEPROTECH), IL-6 (1.000units/mL, PEPROTECH), and PGE_2_(106M/L, Sigma-Aldrich, St Louis, MO, USA) to produce sDCs(8), or αDC1-polarizing cytokine cocktail composed of IL-1ß (25ng/mL), TNF-α (50ng/mL), IFN-α (3.000IU/mL, Intron-A-IFN-α-2b, Schering-Plough International, Kenilworth, NJ, USA), IFN-γ (1.000units/mL, Strathmann Biotech GmbH, Hannover, Germany) and poly(I:C) (20μg/mL, Sigma-Aldrich) to produce αDC1s (11). The DCs were then loaded with the UVB-irradiated T24bladder cancer cell line at a ratio of 2:1 at 2h after the addition of the maturation cytokinesas described previously (16). The matured DCs loaded with dying T24 tumor cells were harvested on day 8, washed, and analyzed by functional assay.

### Preparation of the UVB-irradiated tumor cells as a source of tumor antigen

To load the tumor cells ontoDCs, T24 cells were irradiated with UVB (30mJ/cm^2^) (International Light, Newburyport, MA, USA), cultured overnight in RPMI-1640 (Gibco-BRL) supplemented with FBS to induce apoptosis, and then thoroughly washed. The irradiated tumor cells were loaded onto DCs 2h after the addition of maturation cytokines, according to the previous reports (16). The irradiated dying cells were immediately confirmed using annexin-V and propidium iodide (PI).

### Preparation of bladder cancer cells from patients

Tumor tissues obtained from bladder cancer patients by transurethral resection were minced and lysed for 2-4h at 37ºC in AIM-V medium containing 0.4% collagenase type III. The mononuclear cells were separated by density gradient centrifugation with Ficoll-Hypaque (Lymphoprep) and cryopreserved until their use as target cells in the cytotoxic assay.

### Tumor antigen uptake by DCs

To measure tumor antigen uptake by the DCs, T24 cells were labeled with PKH67-GL- fluorescein isothiocyanate (FITC) (Sigma-Aldrich) before UVB irradiation. After the loading of tumor antigen onto DCs at a ratio of 1:2 on day 6, the αDC1s loaded with dying T24 tumor cells were stained with CD11c-phycoerythrin (PE) and analyzed by flow cytometry for tumor antigen uptake (CD11c^+^/PKH67^+^).

### Immunophenotyping of DCs

To characterize the cell surface phenotypes on DCs, flow cytometry was performed using a FACSAria cell sorter (Becton Dickinson, San Jose, CA, USA) after labeling of the cells with CD86-PE, CD83-FITC, CCR7-FITC (PharMingen, San Diego, CA, USA), and the relevant isotype controls (mouse IgG1 and IgG2a, PharMingen). Cell debris was eliminated from the analysis by forward and side-scatter gating, and the data were analyzed with WinMDI Version 2.9 software (Biology Software Net).

### Cytokine analyses by enzyme-linked immunosorbent assay (ELISA)

The levels of IL-12p70 and IL-10 in the primary culture supernatants of the DCs were measured using Quantikine Immunoassay Kits (R&D Systems, Minneapolis, MN, USA). Additionally, DCs harvested on day 8 were plated in 96-well plates at 2×104 cells/well and stimulated to secrete IL-12 with CD40 ligand (CD40L)-transfected J558 cells (as an analogue of CD40L-expressing Th cells; a gift from Dr. P. Lane, University of Birmingham, UK) at a density of 5×10^4^cells/well. After 24h, the supernatant was harvested and the production of IL-12p70 determined by ELISA (R&D Systems).

### Induction of bladder cancer-specific CTLs

Autologous CD3^+^ (purity >90%) cells were positively isolated from the lymphocyte fraction after Percoll isolation using MACS (Miltenyi Biotec). T cells (1×10^6^cells) were sensitized by autologous αDC1s (1×10^5^ cells) loaded with dying T24 tumor cells. On day 3, rhuIL-2 (5ng/mL, R&D Systems) and IL-7 (10ng/mL, R&D Systems) were added. The CTLs were re-stimulated with the same DCs on day 10. On day 20, the number of antigen-specific T cells was analyzed by IFN-γ enzyme-linked immune spot (ELISPOT) assay. T24 and autologous bladder cancer cells from bladder cancer patients were used as target cells. MHC class I- and II-restricted recognition of the prostate cancer-specific CTLs was analyzed using MHC class I- and II-specific mAbs (clone W6/32 and clone CR3/43, respectively). The ELISPOT data were expressed as the mean number of spots (±SD) per 0.5-2×10^5^ T cells. CTL alone was used as the control.

### Statistical analysis

Data presented are mean plus or minus SD. The statistical significance of differences was assessed using the unpaired t-test. P values <0.05 were considered significant. All statistical analyses were performed with SPSS 17.0 for Windows software (SPSS Inc., Chicago, IL, USA).

## RESULTS

**Figure 1:**
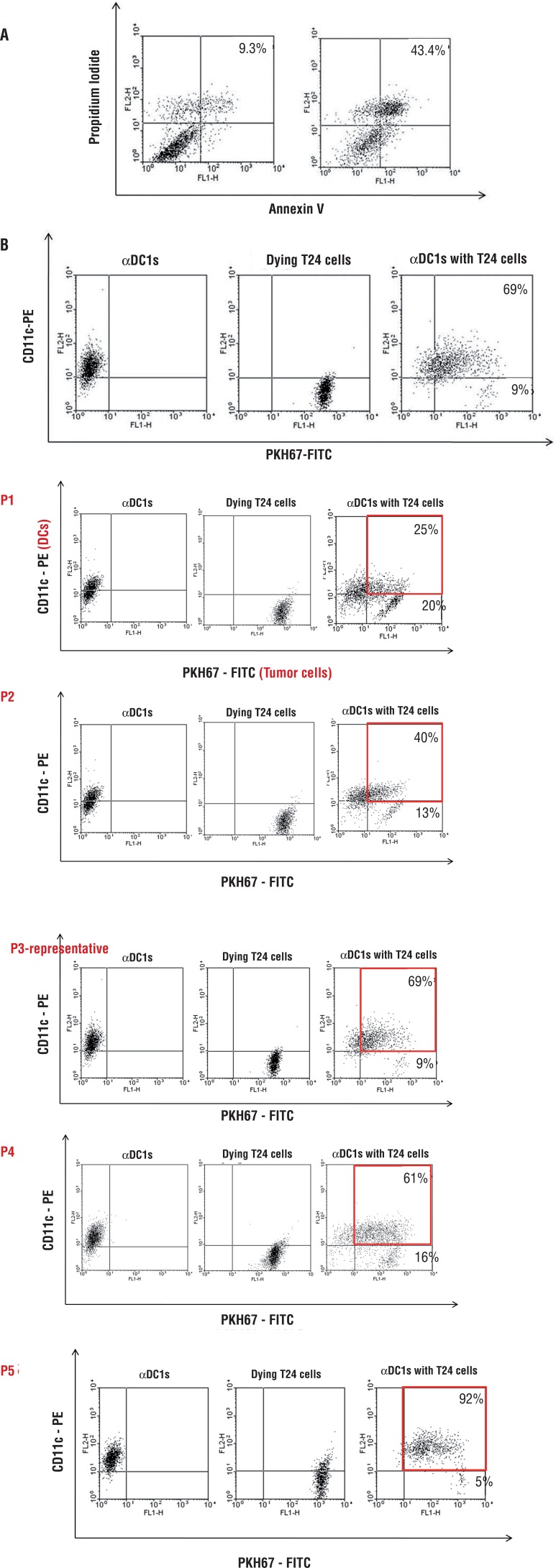
A)Both Annexin-V positive/PI negative cells and Annexin-V/PI double-positive cells were considered to be dying cells;B) After co-culturing, the tumor antigen uptake of the αDC1s was measured by the percentage of double-positive cells. Data are from one representative experiment out of five independent experiments (Data from all cases are provided in supplemental Figures 1 Asupl, Bsupl and Csupl).

Preparation of dying T24 cells as tumor antigens and antigen uptake by αDC1s. About 43.4% of T24 cells were shown as dying cells after UVB irradiation (Figure-1A). As shown in Figure-1B, αDC1s efficiently incorporated the dying T24 tumor antigen (65.8±14.9%; n=5) as measured by flow cytometry (CD11c^+^/PKH-67). The efficacy of antigen uptake ofthe αDC1s and sDCs was similar (data not shown).

### Characteristics of DCs generated from bladder cancer patients

**Figure 2:**
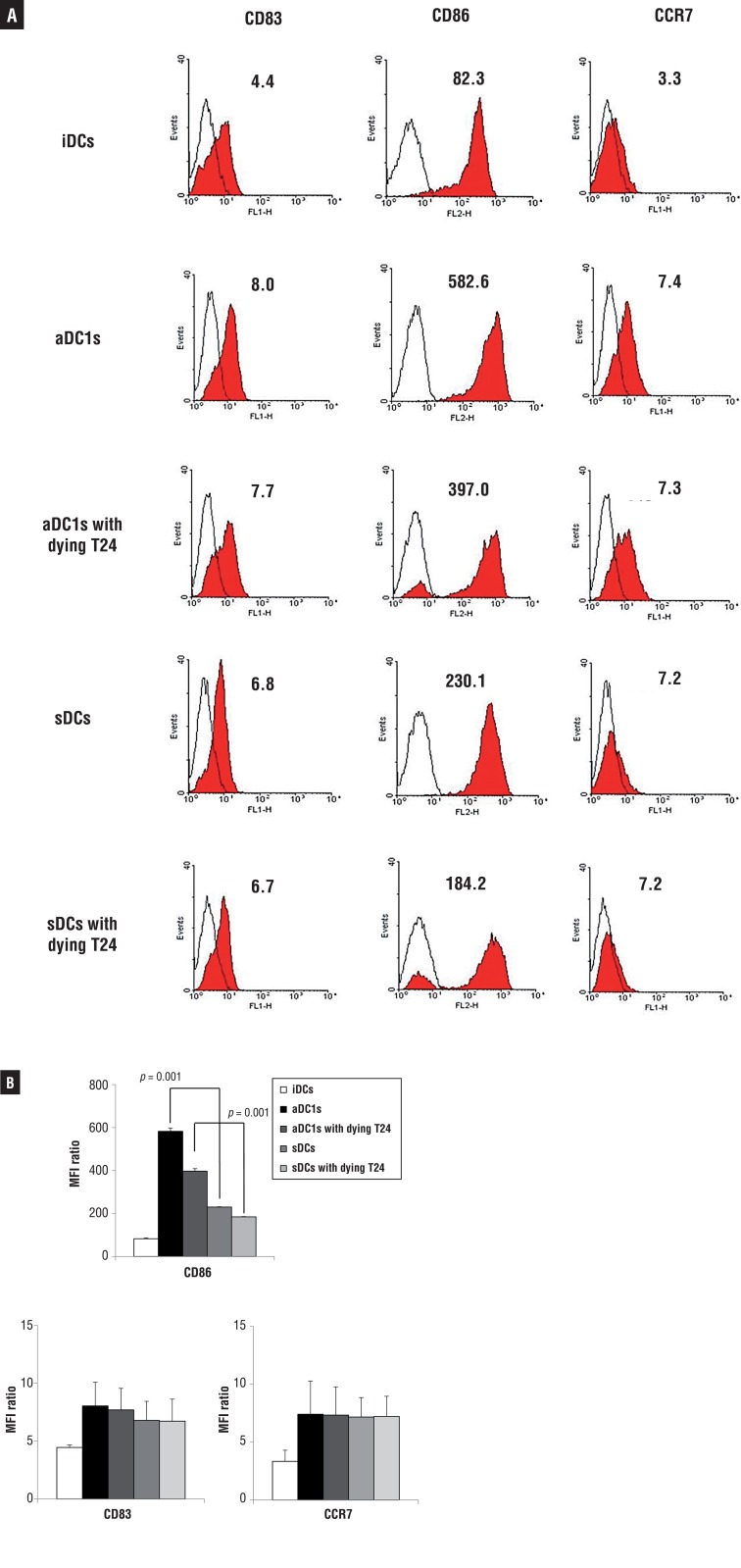
The expression of CD86 was higher in αDC1s than in sDCs (p <0.05). However, there was no difference in the expression of CD83 or CCR7 between the sDCs and αDC1s; A) X axis indicates mean fluorescence intensity (MFI) of FL1 (FITC) or FL2 (PE), and Y axis represent events (strength of fluorescence intensity), respectively;B) shows average MFI values from 3 independent experiments with SD).

The αDC1s showed typical morphology, with large and branching structures aggregated among the cells (not shown). Phenotypically, αDC1s exhibited higher expression of the co-stimulatory molecule (CD86, p=0.001). αDC1s showed comparable expression of the maturation marker (CD83) and predictive marker of migratory ability (CCR7) to the sDCs (Figures2A and 2B).

### IL-12 production of DCs generated from bladder cancer patients

**Figure3:**
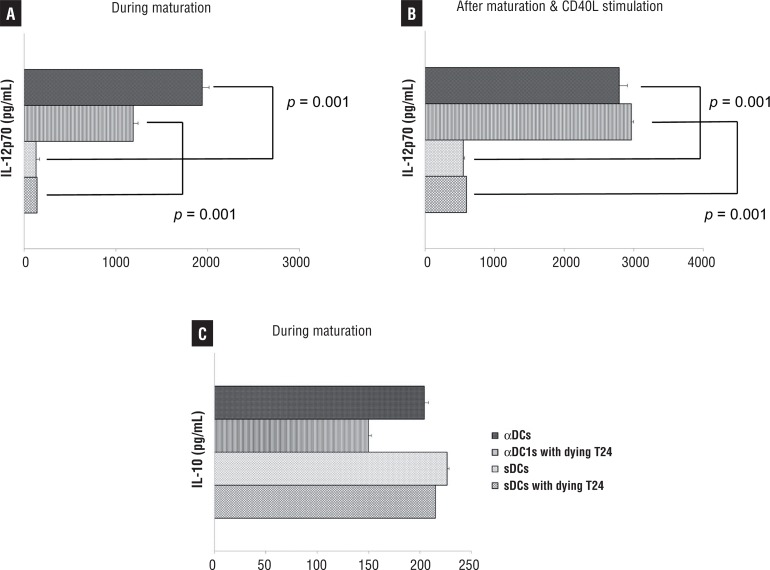
Comparison of cytokine production by DCs loaded with or without tumor cells in A) primary culture supernatant during generation of DCs, and B) after stimulation with CD40L-transfected J558 cells. The αDC1s showed significantly higher production of IL-12p70 during maturation and after stimulation with CD40L-transfected J558 cellsthan the sDCsdid (p<0.05). In addition, production of the inhibitory cytokine IL-10 by αDC1s was not significant.C)Results, expressed as mean (pg/mL) ±SD of triplicate cultures, are representative of five independent experiments.

One of the best ways of determining DC function is to examine cytokine secretion. IL-12p70 is an important cytokine for stimulating naive T cells for Th1 polarization to benefit cancer treatment, but IL-10 is the main inhibitory cytokine for cancer treatment. As shown in Figure-3A, the αDC1s showed higher IL-12p70 levels, as measured in the primary culture supernatant collected at DC harvest (during maturation), than the sDCs did (p=0.001). Furthermore, the αDC1s showed higher production of IL-12p70 after subsequent stimulation with CD40L-transfected J558 cells as compared to the sDCs (p=0.001, Figure-3B). This cytokine secretory capacity of αDC1s was not significantly suppressed by loading tumor antigen. In contrast, production of the inhibitory cytokine IL-10 by αDC1s was not significant (Figure-3C).

### Generation of potent bladder cancer-specific CTLs by autologous DCs loaded with dying tumor cells

**Figure 4:**
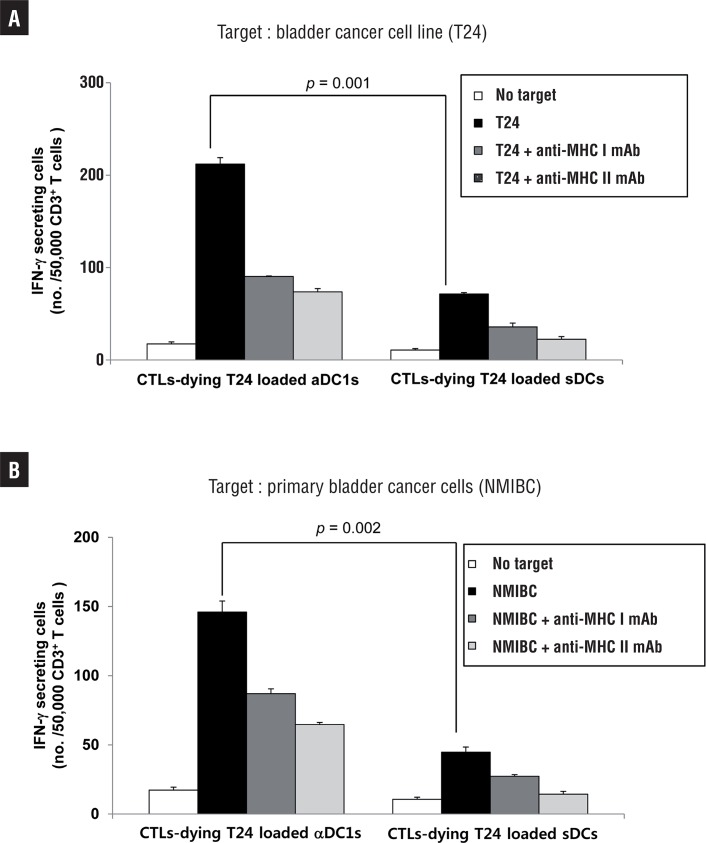
Comparison of bladder cancer-specific CTL induction in vitro with DCs loaded with T24 cells against A)the T24 cell line andB)autologous bladder cancer cells (NMIBC). CD8+ T cells primed by T24 cell-loaded αDC1s showed a larger number of IFN-γ releases than those stimulated by sDCs (p <0.05). ELISPOT data are the mean (±SD) number of IFN-γ-secreting cells of triplicate cultures in three independent experiments.

We next determined the tumor-specific generation of CTLs by DCs. The secretion of IFN-γ by the CTLs was measured in three independent experiments using ELISPOT assay. Even though those results represented the preliminary data in 5 cancer patients, consistent with their high ability to produce IL-12p70, primed CD3^+^ T cells generated by the αDC1s loaded with dying T24 bladder cancer cells showed a larger number of IFN-γ-producing cells against T24 bladder cancer cells (p=0.001, Figure-4A) and autologous bladder cancer cells (NMIBC) obtained from bladder cancer patients than the sDCs did (p=0.002, Figure-4B). The MHC class I- and II-restricted recognition of the CTL response was confirmed using MHC class I- and II-specific mAbs, respectively.

## DISCUSSION

In this study, we investigated the feasibility of cellular immunotherapy using autologous DCs loaded with allogeneic dying bladder cancer cells that could generate potent bladder cancer-specific CTLs against the autologous bladder cancer cells of patients. The αDC1s were successfully generated and significantly increased the expression of several costimulatory molecules by the loading of tumor antigens. Furthermore, αDC1s showed a higher production of IL-12, without significant suppression by tumor antigen loading. In addition, potent bladder cancer-specific CTLs against autologous bladder cancer cells from patients were elicited by autologous αDC1s loaded with dying T24 cells, which was consistent withthe results of previous studies on other cancers (11, 16).

A majority of initial cancer immunotherapy trials have been performed in end-stage cancer patients, and the results of such trials have been disappointing (17). In recent years, there has been a paradigm shift away from administering cancer vaccines to advanced-stage patients and a move toward using cancer vaccines to treat earlier stages of carcinogenesis, before tumor- and treatment-mediated immunosuppressive environments can be established and before the accumulation of mutations that activate redundant pathways for tumor proliferation. Preliminary application of this strategy has yielded promising results. In a transgenic murine model of prostate adenocarcinoma, therapeutic vaccination directed against two different prostate cancer-associated antigens at the earliest stage of carcinogenesis elicited long-term protection against spontaneous prostate cancer development (18). Vaccination of premalignant cervical intraepithelial neoplasia lesions can cause their complete eradication or partial regression to a lower-grade lesion (19). DC-based immunotherapy therapy for NMIBC may fit this modelsince most cases of NMIBC have a reduced tumor burden due to TUR, and a recent study indicated that higher CD3^+^ cell infiltration in NMIBC indicates better cancer-specific survival rates (20). All enrolled male patients in our study were diagnosed withNMIBC.

As mentioned previously, DCs are critical for the presentation of tumor antigens. However, although tumor-infiltrating dendritic cells (TIDC) are present in virtually all human cancers and experimental tumor models, the tumor microenvironment compromises their differentiation, maturation, and survival (21). Any impairment of their normal function may represent a mechanism of tumor immune escape. Several studies demonstrated that DC generation and maturation are inhibited in patients with bladder cancer (22,23). According to Troy et al.(22), a very small proportion of DC in bladder cancer was found to express the activation antigen CD83. In addition, expression of the important costimulatory molecules CD80 and CD86, which are required for activation of T lymphocytes, was restricted. Similarly, Beatty et al.(23) reported that DCs from bladder cancer patient’s urine and tumor tissues showed minimal expression of CD83 and CD86. Therefore, it is reasonable to expect that transplanted functional DCs engineered to overexpress and present specific antigens from bladder tumor cells may be effective in over comingthese dysfunctional autologous DCs. Aside from DC function, identifying effective immunogenic antigens is one of biggest challenges in DC-based vaccine development.

Fry et al.(24) showed that DCs loaded with irradiated apoptotic tumor cells elicited stronger immune responses than tumor cell RNA and tumor lysate. Its major advantage relies on the use of whole tumor proteome, encompassing that way multiple TAAs. Immunogenic cell death induced by UVB-irradiation stimulated the immune system through the release of damage-associated molecular patterns (DAMPs), including the exposure of calreticulins and release of ATP, HMGB1 and heat shock proteins (gp96, HSP70 and HSP90) (25).Based on their results, we used an irradiated dying allogeneic tumor cell line as a tumor antigen. This technique offers the advantages of requiring no knowledge of specific antigens, presenting no obstacles to the preparation of tumor cells, and possessing no need for HLA typing (26).

To enhance DC’s capacity to induce an immune response, a number of immunotherapeutic approaches are being studied at the experimental level. DCs obtained from mice transfected with the Ag85A gene [which has been shown to induce substantial Th cell proliferation and vigorous Th1 cytokine production in humans and mice infected with mycobacterial species, including individuals vaccinated with BCG (27)], which uses murine bladder tumor cell lysate as the source of antigen, exerted enhanced anti-tumor immunity against murine bladder cancer (13). In another study, DC obtained from human umbilical cord blood transfected with secondary lymphoid-tissue chemokine and the interleukin-2 gene, which used established bladder cancer celllines as the source of antigen, enhanced cytotoxicity against same bladder cancer cell lines (28). In addition, Nishiyama et al. (12) reported that autologous DCs pulsed with MAGE-3 peptide-induced autologous CTL in vitro showed tumor regression in pilot clinical trials for advanced bladder cancer.

Our study differs from the above in several important respects. We used DCs primed with dying cells from allogeneic cell lines. In addition to the induction of increased number of tumor-specific T cells, the use of Th1-polarized αDC1s with selectively elevated ability to attract effector (rather than regulatory T cells) and to induce the IL-12-dependent effector (CTL) pathway of differentiation in CD8+ T cells may help to induce tumor-specific immunity of a more desirable pattern than that induced by whole tumor cells or standard non-polarized sDCs (29,30). Additionally, unlikethe Nishiyama study (12), we tested the feasibility of employing αDC1-based vaccines for the treatment of male NMIBC patients with autologous tumor tissue.

In this study, we showed that autologous αDC1s loaded with dying T24 cells could prime T cells to generate potent bladder cancer-specific CTLs thatcould kill bladder cancer cells in maleNMIBC patients. The inhibition of MHC-class molecules by the use of an anti-MHC class antibody prevented the development of CTL responses specific to autologous bladder cancer cells in patients. Thus, the use of an allogeneic tumor cell line as a source of tumor antigens could generate MHC class-restricted T cell responses against autologous bladder cancer cells. However, despite the strong immune data presented in our study, further in vivo and preclinical studies are needed for the development of immunotherapy using αDC1s as a novel treatment for NMIBC.

## CONCLUSIONS

The present study showed that autologous αDC1s loaded with allogeneic dying bladder cancer cells could generate strong bladder cancer-specific CTLs against autologous bladder cancer cells. This technique may offer a highly feasible and effective method for DC-based cancer immunotherapy in male NMIBC patients. Further studies are necessary to enhance the in vivo anti-tumor effect of bladder cancer-specific CTLs generated by autologous dying bladder cancer cells against bladder cancer before clinical application.
